# Potentiality of a triple microRNA classifier: miR-193a-3p, miR-23a and miR-338-5p for early detection of colorectal cancer

**DOI:** 10.1186/1471-2407-13-280

**Published:** 2013-06-08

**Authors:** Fung Lin Yong, Chee Wei Law, Chee Woon Wang

**Affiliations:** 1 Department of Surgery, Faculty of Medicine, University of Malaya, 50603 Kuala Lumpur, Malaysia; 2 Department of Biochemistry, Faculty of Medicine, MAHSA University, 59100 Kuala Lumpur Malaysia

**Keywords:** Colorectal cancer, MicroRNA, MiR-193a-3p, MiR-23a, MiR-338-5p

## Abstract

**Background:**

MicroRNAs (miRNAs) are short, non-coding RNA molecules that act as regulators of gene expression. Circulating blood miRNAs offer great potential as cancer biomarkers. The objective of this study was to correlate the differential expression of miRNAs in tissue and blood in the identification of biomarkers for early detection of colorectal cancer (CRC).

**Methods:**

The study was divided into two phases: (I) Marker discovery by miRNA microarray using paired cancer tissues (n?=?30) and blood samples (CRC, n?=?42; control, n?=?18). (II) Marker validation by stem-loop reverse transcription real time PCR using an independent set of paired cancer tissues (n?=?30) and blood samples (CRC, n?=?70; control, n?=?32). Correlation analysis was determined by Pearson’s test. Logistic regression and receiver operating characteristics curve analyses were applied to obtain diagnostic utility of the miRNAs.

**Results:**

Seven miRNAs (miR-150, miR-193a-3p, miR-23a, miR-23b, miR-338-5p, miR-342-3p and miR-483-3p) have been found to be differentially expressed in both tissue and blood samples. Significant positive correlations were observed in the tissue and blood levels of miR-193a-3p, miR-23a and miR-338-5p. Moreover, increased expressions of these miRNAs were detected in the more advanced stages. MiR-193a-3p, miR-23a and miR-338-5p were demonstrated as a classifier for CRC detection, yielding a receiver operating characteristic curve area of 0.887 (80.0% sensitivity, 84.4% specificity and 83.3% accuracy).

**Conclusion:**

Dysregulations in circulating blood miRNAs are reflective of those in colorectal tissues. The triple miRNA classifier of miR-193a-3p, miR-23a and miR-338-5p appears to be a potential blood biomarker for early detection of CRC.

## Background

Colorectal cancer (CRC) is the third most common cancer worldwide, with an estimation of 1.2 million new cases per year and more than 600,000 deaths [1-3]. The risk of CRC increases with age, whereby most cases are diagnosed in individuals aged 50 and above [4]. Incidence rate of CRC has been increasing in Asian countries. In Malaysia, it ranks the second after lung cancer and breast cancer in men and women, respectively [5]. The 5-year survival rate exceeds 90% when CRC is detected at an early, localized stage [6]. However, most cases are diagnosed at late stages due to inconvenient settings of current CRC screening tests and low population compliance. Colonoscopy has significant contribution in the detection of neoplastic lesions, but the requirements of bowel preparation, sedation and invasive nature have hindered its widespread application as a screening tool [7]. Other structural tests such as computed tomographic colonography and double contrast barium enema are limited by the concern of radiation exposure and cost [8]. Fecal-based analyses such as occult blood, immunochemical and stool DNA tests are common noninvasive screening tests [9]. Nevertheless, they are confined by low sensitivity and specificity against detection of premalignant lesions [10,11]. Although stool DNA test utilizes molecular markers such as *KRAS*, *APC* and *TP53* genes as detection targets, it is not favorably recommended by the US Preventive Service Task Force due to high cost, uncertainty of test performance and labor-intensive handling [12-14]. Thus, there is an imperative need for other noninvasive biomarkers to complement and improve current diagnostic and prognostic tools in CRC.

MicroRNAs (miRNAs) are short (19–22 nucleotides), non-coding RNA molecules that act as regulators of gene expression [15]. Although their main mechanism of action is through mRNA degradation or translational inhibition, they can also induce gene activation [16,17]. The pioneer discovery of miRNAs was initiated by Lee and colleagues in 1993 when they found lin-4, a small RNA molecule that was capable of controlling the larval development of *Caenorhabditis elegans*[18]. Since then, a vast number of miRNAs have been reported. To date, there are more than 2000 entries of human miRNAs in the miRNA database, miRBase 19.0, constituting around 1% of total genes that can regulate up to one third of human genome [19]. MiRNAs are evolutionary conserved across species and expressed in a tissue-specific manner [20]. They have been determined to play important roles in cancer pathophysiology such as cell proliferation, differentiation, apoptosis and metastasis [21-23]. MiRNA genes are frequently located at fragile sites and genomic regions of deletion and amplification implicated in cancers [24,25]. They can confer both oncogenic and tumor suppressive roles, depending upon their downstream targets [26-28]. MiRNAs are detectable in tissues, blood, feces and other body fluids such as saliva, tears and urine [29-32]. The presence of miRNAs in the circulation system is mediated via exosome transfer to the surrounding microenvironment [33,34]. Blood-based miRNA profiling has become a favorable area of research for the identification of noninvasive biomarkers in cancers and other diseases [35-38]. In addition, a study by Heneghan *et al.* reported a strong preference of using whole blood to serum or plasma for systemic miRNAs detection and quantification [39]. Circulating blood miRNAs are generally Ago-bound and protected from endogenous RNases that enable them to serve as stable blood biomarkers [40,41].

An important concern for utilization of miRNAs as biomarkers is whether the dysregulated miRNAs are related to CRC alone or as a general mechanism in histologic progression to cancer [29,42]. The objective of this study was to correlate the differential expression of miRNAs in tissue and blood in the identification of biomarkers for early detection of CRC.

## Methods

### Study design and sample selection

A case–control study was designed to identify blood miRNAs that are reflective of those in colorectal tissues. This study was performed with the approval from Medical Ethics Committee of University of Malaya Medical Centre (UMMC) (reference number 805.9). A total of 162 participants were enrolled from January 2011 to January 2013 at UMMC. A number of 112 blood samples and a subset of 60 paired cancer tissues with adjacent normal mucosa were collected from primary CRC patients. The histology was confirmed by pathological analysis and staged according to the tumor-node-metastasis (TNM) staging system of the International Union Against Cancer. For the control group, 50 blood samples were collected from individuals who were proven to be colonic disease-free after colonoscopy. They were matched to the CRC patients according to age, gender and race. Written informed consent has been obtained from each participant. The tissue and whole blood samples were collected in tubes containing RNA*later* (Ambion, Austin, TX).

### Total RNA isolation

Total RNA (including miRNAs) from tissue and blood samples were extracted using Qiagen miRNeasy Mini Kit (Qiagen, Valencia, CA) and Ribopure Blood RNA Isolation Kit (Ambion) respectively, according to manufacturer’s instructions. RNA concentration and integrity were determined using NanoDrop 2000 Spectrophotometer (Thermo Scientific Wilmington, DE) and Agilent 2100 Bioanalyzer (Agilent Technologies, Santa Clara, CA). RNA samples with the RNA integrity number?≥?7.0 and the absence of DNA contamination were used for downstream experiments [43].

### MiRNA microarray expression profiling and analysis

The miRNA expression profiles were generated by GeneChip miRNA 2.0 Array (Affymetrix, Santa Clara, CA). This array contains 15,644 probe sets, covering 131 organisms and detecting 1,105 human mature miRNAs. The content is derived from Sanger miRBase miRNA database version 15.0. Briefly, 1 μg of total RNA was biotin-labeled using 3DNA Array Detection Flashtag Biotin HSR RNA Labeling Kit (Genisphere LLC, Hatfield, PA). The samples were hybridized overnight in Affymetrix Hybridization Oven 640, washed and stained using Affymetrix Fluidics Station 450 and scanned with GeneChip Scanner 3000 7G. Cell intensity file was generated in the GeneChip Command Console software and used for further analysis with GeneSpring GX 12.0 (Agilent Technologies). Robust multichip averaging (RMA) algorithm was applied for background correction and probe summarization of perfect match values in each microarray chip. Median intensity values for each miRNA from the same replicates were calculated and subjected to quantile normalization to normalize the data across different arrays [44]. The normalized data were analyzed using *t*-test/ANOVA analysis with *p* value computations done asymptotically at *p*?<?0.05. Subsequently, the gene lists were filtered at a fold change cut-off of 1.5. Hierarchical clustering was computed using similarity measure of Euclidean distance and average linkage rule and expressed in the form of heat map and three dimensional (3D) principal component analysis (PCA) plot. The miRNA microarray data reported are MIAME compliant and have been submitted to the NCBI Gene Expression Omnibus (GEO) database (Accession: GSE39845).

Two independent miRNA microarray profiling studies of tissue and blood were conducted. In tissue miRNA array, 30 paired cancer tissue and the adjacent normal mucosa samples were pooled according to stages II (n?=?10), III (n?=?10) and IV (n?=?10), respectively. In blood miRNA array, blood samples from 42 CRC cases were grouped by tumor location (colon; rectum) and pooled into stages I (n?=?3; n?=?3), II (n?=?9; n?=?3), III (n?=?9; n?=?3) and IV (n?=?9; n?=?3). Blood samples from 18 healthy controls were used for the profiling study. Due to limited availability of stage I CRC cases, only one replicate was performed for both colon and rectal samples. Similarly, the profiling analyses of rectal samples for stages II, III and IV were also performed in one replicate. On the other hand, the profiling analyses of stages II, III and IV of colon samples were performed in triplicates and control samples in six replicates, with n?=?3 each. The blood samples were obtained from the same group of patients who have donated their tissue samples. The tissue and blood miRNA profiles were then correlated and used to determine the miRNAs that were concurrently expressed.

### Stem-loop reverse transcription real time PCR (RT-PCR) assay

The miRNA microarray results were validated with stem-loop RT-PCR using Taqman MicroRNA Assay on StepOnePlus Real Time PCR system (Applied Biosystems, Foster City, CA). An independent set of 30 paired cancer tissues (stage II, n?=?10; stage III, n?=?10; stage IV, n?=?10), 70 blood samples from CRC patients (stage I, n?=?19; stage II, n?=?20; stage III, n?=?19; stage IV, n?=?12) and 32 blood samples from healthy controls were used in the validation study. This is a two-step protocol which utilizes reverse transcription with miRNA-specific primer followed by quantitative real time PCR with Taqman probe. Seven miRNAs were selected for this purpose and the primer sequences are listed in Additional file 1. Briefly, total RNA of 10 ng was subjected to reverse transcription using Taqman MicroRNA RT Kit which comprised of 100 mM dNTPs, 50 U/ul Multiscribe Reverse Transcriptase, 10X RT Buffer and 20 U/ul RNase Inhibitor (Applied Biosystems). The RT product was then diluted at 1:15 dilution and added to the reaction mixture of Taqman 2X Fast Advanced Master Mix and Taqman 20X MicroRNA Assay for RT-PCR. All assays were performed in triplicate and adhered to the protocols provided by the manufacturer. The expression of each miRNA relative to RNU48 as endogenous control was presented as ∆CT. A Ct value of 35 was set as the cut-off value for defining as non-detected [45,46]. Fold change was determined using comparative CT (2^-∆∆CT^) method [47].

### Statistical analysis

The patients’ demographics were reported as mean?±?standard deviation or frequencies and percentages for continuous and categorical variables, respectively. Microarray data analysis was carried out as outlined above. T-tests were used to determine the level of significance (*p*?<?0.05) of the selected miRNAs. Correlation analysis was determined by Pearson’s test. Logistic regression and receiver operating characteristics (ROC) curve analyses were applied to obtain diagnostic utility of the miRNAs. Statistical analysis was performed using IBM SPSS version 16.0 software (IBM Corporation, Armonk, NY).

## Results

### Demographics study

A total of 112 CRC patients and 50 healthy controls were enrolled in this study (Table 1). No significant differences were observed between the CRC patients and controls in the distribution of age (*p*?=?0.071, chi-square test) and gender (*p*?=?0.174, Fisher’s exact test). Malaysia is comprised of a multi-ethnic population. The National Cancer Registry of Malaysia has reported a higher proportion of CRC cases in the Malaysian Chinese population [5]. Thus, approximately 52% of the samples obtained were from the Malaysian Chinese and the remainders were from the Malays and Malaysian Indians (*p*?=?0.202, Fisher’s exact test). All CRC cases in this study were adenocarcinomas. The characteristics of the subset of 60 paired cancer tissues with adjacent normal mucosa were listed in Table 2.

**Table 1:** Patients’ demographics: characteristics of CRC patient and control blood cohorts

**Characteristics**		**CRC patient blood cohort**	**Control blood cohort**	***p*value**^**a**^
		**(n?=?112), n (%)**	**(n?=?50), n (%)**	
Average age (years)		64.4?±?9.0	61.5?±?9.3	0.071
Gender	Male	67 (59.8%)	24 (48.0%)	0.174
	Female	45 (40.2%)	26 (52.0%)	
Race	Malay	26 (23.2%)	15 (30.0%)	0.202
	Chinese	64 (57.1%)	21 (42.0%)	
	Indian	22 (19.6%)	14 (28.0%)	
TNM stage	I	25 (22.3%)		
	II	32 (28.6%)		
	III	31 (27.7%)		
	IV	24 (21.4%)		
Tumor location	Colon	72 (64.3%)		
	Rectum	40 (35.7%)		
Tumor grading	G1	25 (22.3%)		
(adenocarcinoma)	G2	76 (67.9%)		
	G3	11 (9.8%)		

^a^*p* value for age was calculated by chi-square test; *p* values for gender and race were calculated by Fisher’s exact test.

**Table 2:** Patients’ demographics: characteristics of the subset of paired cancer tissue from CRC patients

**Characteristics**		**Subset of paired cancer tissue**
		**(n?=?60), n (%)**
Average age (years)		63.8?±?10.6
Gender	Male	33 (55.0%)
	Female	27 (45.0%)
Race	Malay	10 (16.7%)
	Chinese	38 (63.3%)
	Indian	12 (20.0%)
TNM stage	I	0
	II	20 (33.3%)
	III	20 (33.3%)
	IV	20 (33.3%)
Tumor location	Colon	60 (100.0%)
	Rectum	0
Tumor grading	G1	13 (21.7%)
(adenocarcinoma)	G2	43(71.7%)
	G3	4 (6.7%)

### Tissue and blood miRNA microarray profiling

**Figure 1:**
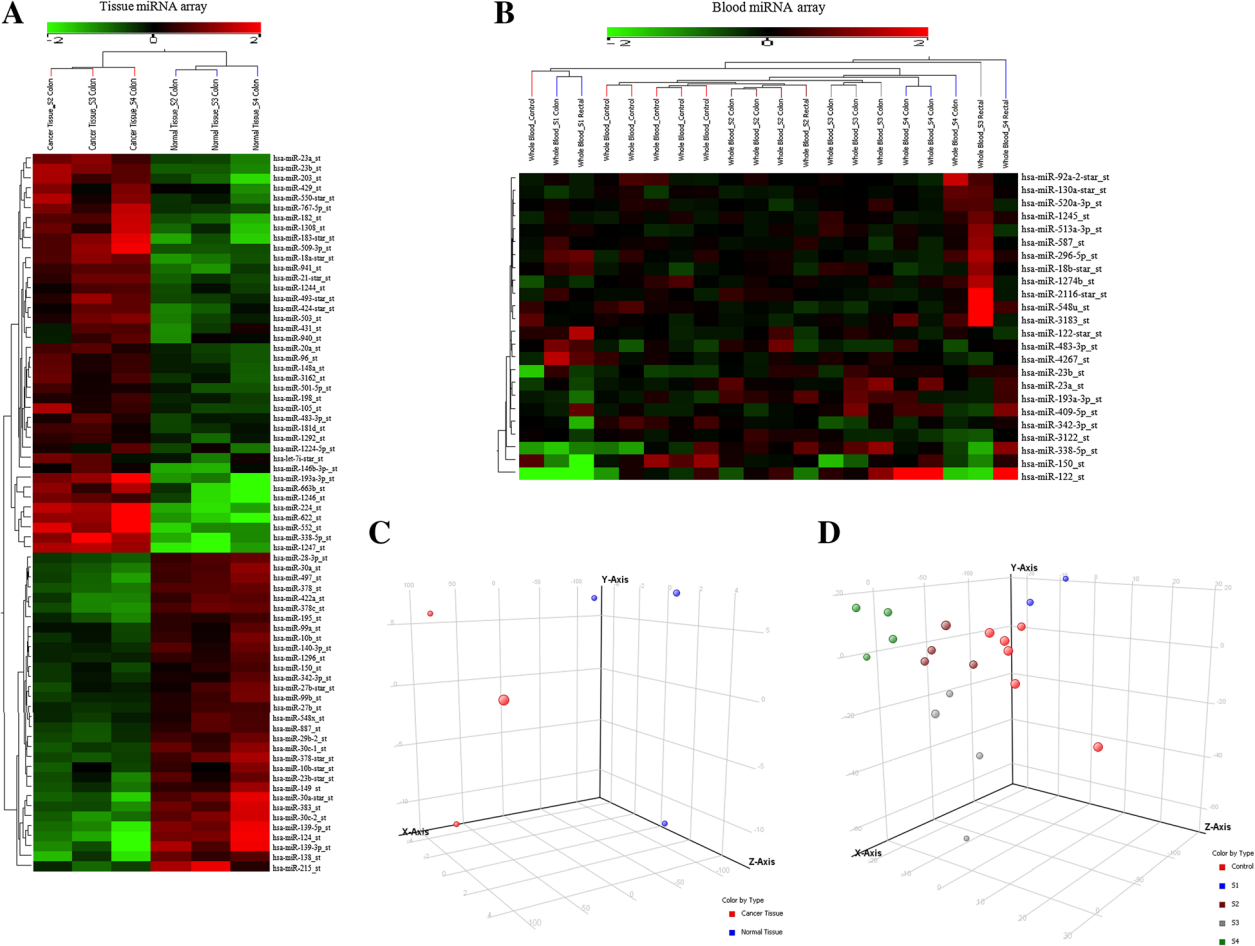
**Hierarchical clustering and PCA analyses.** (**A**) Tissue miRNA array. Data were expressed as fold change of cancer tissue versus adjacent normal mucosa. (**B**) Blood miRNA array. Data were expressed as fold change of TNM stages (I, II, III and IV) versus healthy controls. S1, stage I; S2, stage II; S3, stage III; S4, stage IV. Green: down-regulation; red: up-regulation. (**C**) PCA plot of paired cancer tissue and adjacent normal mucosa samples. Color by type, red: cancer tissue; blue: adjacent normal mucosa. (**D**) PCA plot of whole blood samples. Color by type, red: control; blue: stage I; brown: stage II; grey: stage III; green: stage IV.

In the discovery of global miRNA expression in cancer tissue and whole blood, two independent miRNA profiles were generated. Hierarchical clustering analyses of the tissue and blood arrays were shown in Figures 1A and 1B, respectively. The heat maps indicated the number of miRNAs that were differentially regulated between the normal/control group versus the cancer group. Moreover, differential expressions of the miRNAs were observed among samples of different TNM staging. Many miRNAs that were poorly expressed in normal/control samples have been determined to be highly expressed in CRC samples. At the fold change cut-off of 1.5, the tissue miRNA array revealed 40 significantly up-regulated miRNAs and 32 down-regulated miRNAs (Additional file 2A). On the other hand, the blood miRNA array only revealed 15 significantly up-regulated and 9 down-regulated miRNAs (Additional file 2B). Next, 3D PCA plots were computed to provide visual representations of the samples. The 3D PCA plot distributes the samples into a three dimensional space based on variance in miRNA gene expressions. Samples from the same group and stage were found to be clustered together in distinctive patterns, as shown in the Figure 1C and 1D, respectively.

**Figure 2:**
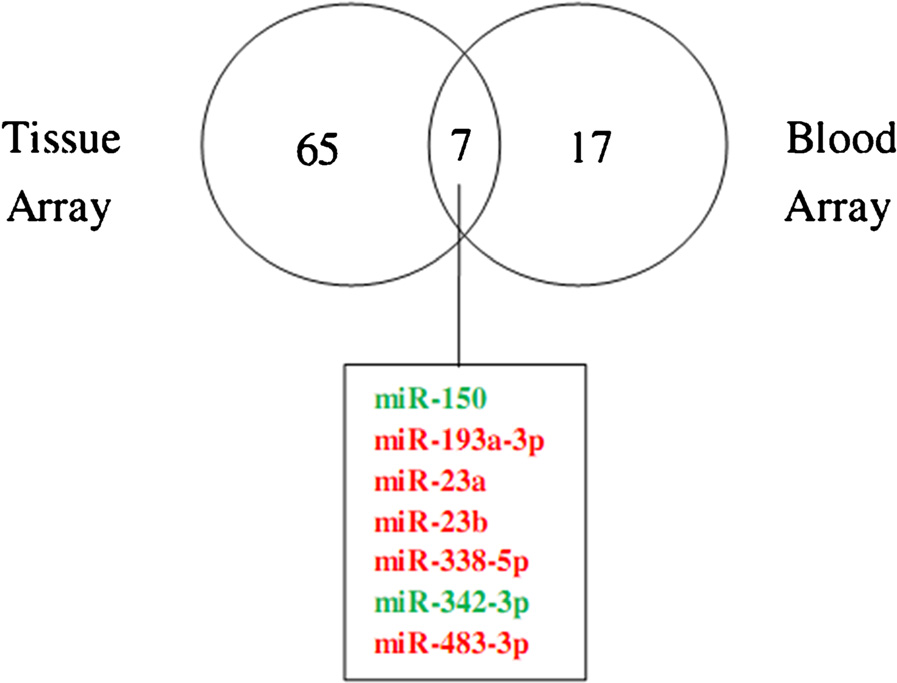
**Venn diagram of differentially expressed miRNAs in tissue and blood arrays.** Down-regulated miRNAs were indicated in green while up-regulated miRNAs were indicated in red.

The tissue array revealed a higher number of deregulated miRNAs and those that were concurrently expressed in blood array were selected for further validation (Figure 2). The selected miRNAs consisted of two down-regulated (miR-150 and miR-342-3p) and five up-regulated miRNAs (miR-193a-3p, miR-23a, miR-23b, miR-338-5p and miR-483-3p).

### Validation of miRNA expressions by RT-PCR

The selected miRNAs from miRNA profiling were validated with RT-PCR using an independent set of tissue and blood samples. RNU48 was chosen as the endogenous control in data normalization and its expression was found to be stable and reproducible. No significant difference was found in the level of RNU48 (*p*?=?0.483) between CRCs and controls. Using a new subset of 30 paired cancer tissue and adjacent normal mucosa samples, significant deregulations were noticed in the panel of seven miRNAs using paired *t*-test (*p*?=?0.039 for miR-150, *p*?=?0.037 for miR-193a-3p, *p*?=?0.031 for miR-23a, *p*?=?0.025 for miR-23b, *p*?=?0.023 for miR-338-5p, *p*?=?0.025 for miR-342-3p, *p*?=?0.009 for miR-483-3p). For blood samples validation, significant elevations were detected in the levels of miR-193a-3p (*p*?<?0.001), miR-23a (*p*?=?0.043), miR-23b (*p*?=?0.045), miR-338-5p (*p*?<?0.001) and miR-483-3p (*p*?=?0.010) in CRC cases. However, no significant difference was observed in the levels of miR-150 (*p*?=?0.450) and miR-342-3p (*p*?=?0.560). The absolute Ct values of miRNAs in both tissue and blood samples ranged from 21 to 28 for miR-150, 27 to 33 for miR-193a-3p, 23 to 30 for miR-23a, 23 to 31 for miR-23b, 28 to 33 for miR-338-5p, 25 to 29 for miR-342-3p and 26 to 33 for miR-483-3p.

### Relationship between tissue and blood miRNAs

**Figure 3:**
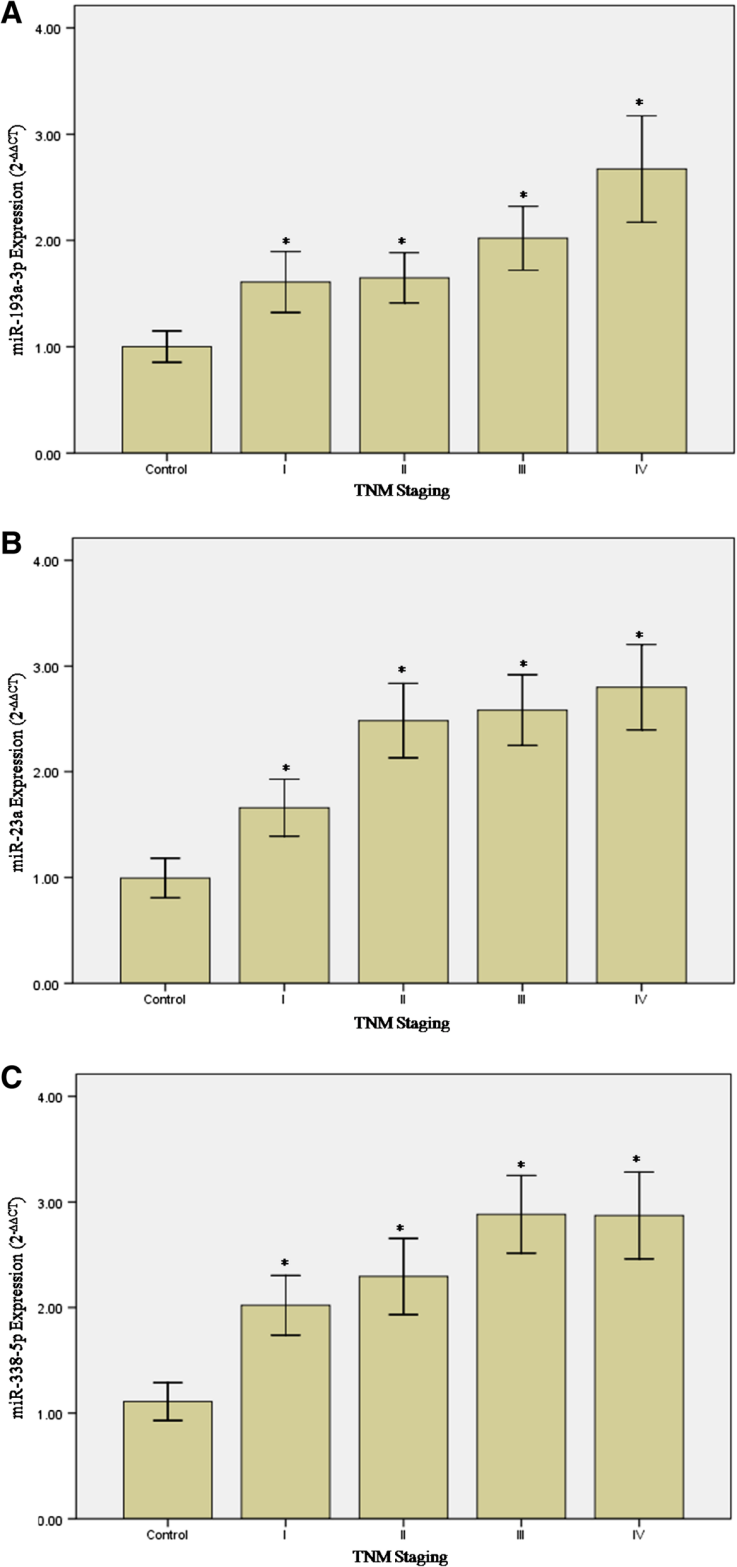
**Expression levels of circulating blood (A) miR-193a-3p, (B) miR-23a and (C) miR-338-5p.** An increasing trend of expression was observed in the circulating blood miRNAs in the more advanced stages when compared with controls. Statistically significant differences were tested at *p?<?0.05. MiRNA expression was presented as fold change (2^-∆∆CT^); error bars represent mean?±?SEM.

Given that miR-193a-3p, miR-23a, miR-23b, miR-338-5p and miR-483-3p were significantly up-regulated in both tissue and blood samples from the validation study, we have proceeded to investigate the correlation between them. The purpose is to provide a stronger confirmation that deregulated miRNA expressions in the systemic circulation are potential indicators of what is happening at tissue level. Controlling for age, gender, race and TNM staging, correlation analyses between tissue and blood RT-PCR data for miR-193a-3p (r?=?0.811, *p*?<?0.001), miR-23a (r?=?0.827, *p*?<?0.001), miR-23b (r?=?0.044, *p*?=?0.818), miR-338-5p (r?=?0.831, *p*?<?0.001) and miR-483-3p (r?=?0.373, *p*?=?0.042) were conducted. The results indicated significant positive correlations in the levels of miR-193a-3p, miR-23a and miR-338-5p between tissue and blood samples. MiR-23b was not significantly correlated while miR-483-3p revealed weak correlation. Thus, miR-193a-3p, miR-23a and miR-338-5p were selected as the triple miRNA classifier in our study. Moreover, an increasing trend of expression was observed in these circulating blood miRNAs from the less advanced stages (I and II) to the more advanced stages (III and IV) when compared with controls (*p*?<?0.05) (Figure 3A, 3B and 3C).

### Diagnostic value of the triple miRNA classifier: miR-193a-3p, miR-23a and miR-338-5p in CRC

**Figure 4:**
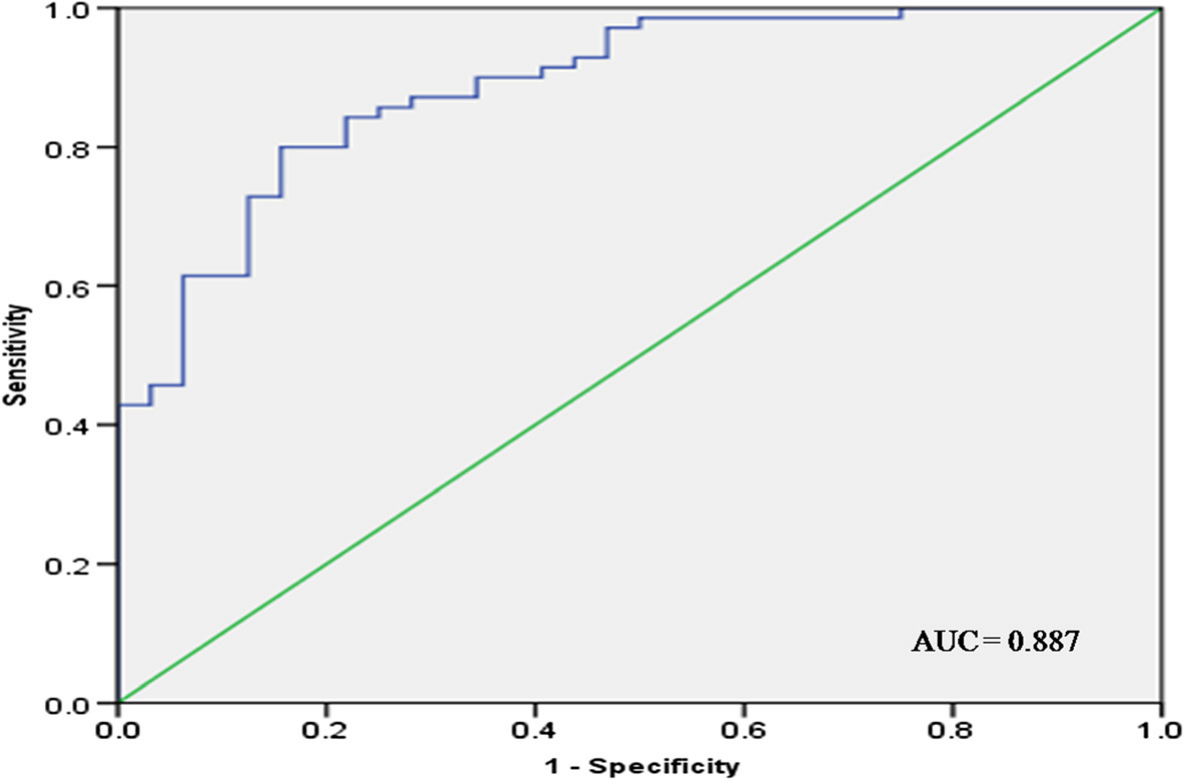
**ROC curve analysis using the triple miRNA classifier of miR-193a-3p, miR-23a and miR-338-5p.** The triple miRNA classifier yielded an AUC of 0.887 (95% CI: 0.821 - 0.953) with 80.0% sensitivity, 84.4% specificity and 83.3% accuracy.

The predictive performance of individual circulating blood miRNA and the triple miRNA classifier for defining CRC were demonstrated by multivariate logistic regression analysis (Table 3). The triple miRNA classifier of miR-193a-3p, miR-23a and miR-338-5p gave the best performance and could be a potential biomarker in the detection of CRC. The optimal cut-off value for sensitivity and specificity was determined based on the highest Youden’s Index in ROC curve analysis [48]. The triple classifier has a stronger differentiation power than individual or double combination of miRNAs. The classifier has an increased area under ROC curve (AUC) of 0.887 (confidence interval [CI]: 0.821 – 0.953) with 80.0% sensitivity, 84.4% specificity and 83.3% accuracy, illustrating an improved diagnostic value of these triple combination of miRNAs (Figure 4).

**Table 3:** Multivariate logistic regression analysis of individual blood miRNA and miRNA classifier of miR-193a-3p, miR-23a and miR-338-5p

**Statistical parameters**	**miR-193a-3p**	**miR-23a**	**miR-338-5p**	**miR-193a-3p?+?miR-23a**	**miR-193a-3p?+?miR-338-5p**	**miR-23a?+?miR-338-5p**	**miR-193a-3p?+?miR-23a?+?miR-338-5p**
AUC	0.852	0.787	0.871	0.852	0.872	0.873	0.887
95% CI	0.768 - 0.935	0.685 - 0.889	0.800 - 0.942	0.770 - 0.935	0.798 - 0.946	0.803 - 0.942	0.821 - 0.953
Sensitivity (%)	100.0	94.3	81.4	74.3	94.3	81.4	80.0
Specificity (%)	56.2	53.1	75.0	81.2	65.6	75.0	84.4
Accuracy (%)	83.3	81.4	83.3	83.3	83.3	82.4	83.3
Youden’s Index^a^	0.562	0.474	0.564	0.555	0.599	0.564	0.644
Cut-off value	0.346	0.502	0.640	0.745	0.474	0.636	0.728

^a^Youden’s Index?=?sensitivity?+?specificity - 1.

## Discussion

MiRNA profiling studies in CRC and other diseases have been vastly reported [35-38]. For instance, a recent tissue miRNA profiling study by Piepoli *et al.* revealed a cluster of dysregulated miRNAs (miR-195, miR-1280, miR-140-3p and miR-1246) that could be used to distinguish between colorectal and pancreatic cancers [49]. An investigation by Hamfjord *et al.* using high throughput sequencing in paired cancer tissues has reported 16 novel miRNAs (miR-490-3p, miR-628-3p/-5p, miR-1297, miR-3151, miR-3163, miR-3622a-5p, miR-3656, miR-105, miR-549, miR-1269, miR-1827, miR-3144-3p, miR-3177, miR-3180-3p and miR-4326) that have not been previously described in CRC [50]. For blood-based miRNA profiling, Ahmed *et al.* have identified a huge panel of differentially expressed miRNAs in sporadic colon cancer patients [51]. Our data corresponded to theirs whereby an overlap of certain markers (miR-130a, miR-150, miR-18b, miR-338, miR-342 and miR92a) was observed in our blood array study. Apart from that, Ng *et al.* have evaluated a panel of 95 miRNAs using real-time PCR-based array and plasma miR-17-3p and miR-92 were shown to be significantly expressed between the CRC cases and controls [52]. The main aspect of our study is to determine whether the circulating blood miRNAs are reflective of those in the tissues. Therefore, we have investigated the correlation of miRNA expressions between paired cancer tissue and whole blood of CRC patients. The levels of miR-193a-3p, miR-23a and miR-338-5p were significantly up-regulated and positively correlated in both the tissue and blood samples. The triple miRNA classifier identified in this study was of high sensitivity (80.0%), specificity (84.4%) and accuracy (83.3%) in defining CRC. The findings have collectively implicated the potentiality of circulating blood miRNAs as noninvasive biomarkers.

Mir-193a-3p is part of the miR-193 family, together with miR-193a-5p and miR-193b. Nevertheless, the information on miR-193a-3p is limited and its molecular mechanism and role in carcinogenesis remain largely unknown. To our knowledge, the present paper is the first report on the up-regulation of miR-193a-3p in CRC. Although miR-193a-3p has not been mentioned in CRC, its dysregulation has been observed in pleural mesothelioma, ostersarcoma and oral cancer, suggesting its impact in tumorigenesis [53-55]. Clearly, the role of miR-193a-3p in cancer needs to be robustly investigated.

The role of miR-23a has been reported with varying conclusions. Recently, Jahid *et al.* revealed the function of miR-23a in promoting the migration and invasion of CRC cells and stem cells by down-regulating *metastasis suppressor 1 (MTSS1)* gene [56]. The finding is consistent with our study whereby there is an increasing expression of miR-23a in the more advanced tumors. A deep sequencing project using serum samples has demonstrated a common up-regulation of miR-23a in CRC and lung cancer patients [57]. An *in vitro* study by Zhu *et al.* using gastric adenocarcinoma cell line (MGC803) revealed a growth-promoting function of miR-23a via regulation of *interleukin-6 receptor (IL-6R)* gene [58]. Besides its individualistic function, miR-23a has been shown to possess cooperative functions with miR-27a and miR-24 [59]. The three miRNAs are derived from a single primary transcript, located on chromosome 9q22. These miRNAs have been bioinformatically predicted to regulate cell proliferation and tissue development via Wnt signaling pathway [60]. Wnt pathway is associated with advancement of dysplasia in aberrant crypt foci and acts as ‘gatekeeper’ in the initiation of adenoma-carcinoma sequences in CRC. In addition, Rogler *et al.* found miR-23b, a paralog of miR-23a to have an amplification effect with miR-23a in regulating transforming growth factor beta signaling by targeting *SMAD* genes [61]. This is in support with our microarray and validation studies whereby mir-23a and miR-23b were discovered to be significantly up-regulated in both tissue and blood samples. MiR-23a, together with miR-23b have been reported to play certain roles in glutamine catabolism, cell cycle regulation and glucose metabolism via regulation of *c-Myc* gene [59,62]. On the contrary, miR-23a expression has also been found to be repressed in several malignancies, including oral squamous cell carcinoma and acute promyelocytic leukemia [55,63]. The findings unveiled the possibility of diverse miRNA functions in different cell types and diseases.

MiR-338-3p and miR-338-5p are isoforms of miR-338. In general, over-expression of miR-338 has been observed in many cancers, namely CRC, hepatocellular carcinoma and head and neck/oral cancer [64-66]. A tissue miRNA profiling by Schetter *et al.* revealed 37 miRNAs, including miR-338, to be differentially expressed in CRC tissues when compared with paired noncancerous tissues [64]. In a recent investigation on CRC recurrence-related miRNAs by Ju *et al.*, miR-338-5p was found to be significantly up-regulated and positively correlated with cancer metastasis [67]. Our findings corresponded to their study whereby mir-338-5p expression was shown to be significantly higher in the more advanced tumors.

An individual miRNA is definitely not an appealing marker in discriminating CRC. The fact that miRNAs could act as cell proliferating factors in certain cancers and apoptotic factors in another has evoked the necessity of studying their cooperative functions in order to provide a holistic picture of miRNA regulations in cancer biology and pathogenesis.

## Conclusion

Dysregulations in circulating blood miRNAs are reflective of those in colorectal tissues. The triple miRNA classifier of miR-193a-3p, miR-23a and miR-338-5p appears to be a potential blood biomarker for early detection of CRC. Our study serves as an exploratory basis for further investigation in larger prospective and randomized clinical studies with higher number of samples from healthy controls and patients of advanced adenoma and various stages of CRC. A noninvasive miRNA screening assay using the triple miRNA classifier could then be developed to identify asymptomatic individuals with colorectal neoplasia prior to more invasive colonoscopy examination.

## Abbreviations

3D: Three dimensional; AUC: Area under ROC curve; CI: Confidence interval; CRC: Colorectal cancer; GEO: Gene Expression Omnibus; IL-6R: Interleukin-6 receptor; miRNAs: microRNAs; MTSS1: Metastasis suppressor 1; PCA: Principal component analysis; RMA: Robust multichip averaging; ROC: Receiver operating characteristics; RT-PCR: Reverse transcription real time PCR; TNM: Tumor-node-metastasis; UMMC: University of Malaya Medical Centre.

## Competing interests

The authors declare that they have no competing interests.

## Authors’ contributions

FLY has carried out the experiments and drafting of the manuscript. CWL was responsible for collection of samples and clinical interpretations. CWW was responsible for overall supervision. All authors contributed equally in study design, data analysis and have approved the final manuscript.

## Pre-publication history

The pre-publication history for this paper can be accessed here:

http://www.biomedcentral.com/1471-2407/13/280/prepub

## Supplementary Material

Additional file 1 **Taqman 20X MicroRNA Assays.** Reporter dye: FAM; reporter quencher: NFQ (Applied Biosystems). Click here for file

Additional file 2 **(A) List of significantly deregulated miRNAs (*p*<0.05) from tissue miRNA array.** MiRNA expression was shown as fold change of cancer tissue versus adjacent normal mucosa. Positive value denotes up-regulation and negative value denotes down-regulation. (**B**) List of significantly deregulated miRNAs (*p*?<?0.05) from blood miRNA array. MiRNA expression was shown as fold change of TNM stages (I, II, III and IV) versus control samples. Positive value denotes up-regulation and negative value denotes down-regulation. Click here for file
