# Supplementary material for: Potentiality of a triple microRNA classifier: miR-193a-3p, miR-23a and miR-338-5p for early detection of colorectal cancer
Source: BMC Cancer. 2013 Jun 8;13:280. doi: 10.1186/1471-2407-13-280 (PMC3691634; doi:10.1186/1471-2407-13-280)
Supplement: Additional file 1 — Taqman 20X MicroRNA Assays. Reporter dye: FAM; reporter quencher: NFQ (Applied Biosystems). [file 1471-2407-13-280-S1.doc]

**Additional file 1**

**Taqman 20X MicroRNA Assays.** Reporter dye: FAM; reporter quencher: NFQ (Applied Biosystems).

| **Assay Name** | **Assay ID** | **Context Sequence** | **Mature miRNA Accession** |
| --- | --- | --- | --- |
| hsa-miR-23a | 000399 | AUCACAUUGCCAGGGAUUUCC | MIMAT0000078 |
| hsa-miR-23b | 000400 | AUCACAUUGCCAGGGAUUACC | MIMAT0000418 |
| hsa-miR-150 | 000473 | UCUCCCAACCCUUGUACCAGUG | MIMAT0000451 |
| hsa-miR-193a-3p | 002250 | AACUGGCCUACAAAGUCCCAGU | MIMAT0000459 |
| hsa-miR-483-3p | 002339 | UCACUCCUCUCCUCCCGUCUU | MIMAT0002173 |
| hsa-miR-338-5p | 002658 | AACAAUAUCCUGGUGCUGAGUG | MIMAT0004701 |
| hsa-miR-342-3p | 002260 | UCUCACACAGAAAUCGCACCCGU | MIMAT0000753 |
| RNU48 | 001006 | GATGACCCCAGGTAACTCTGAGTGTGTCGCTGATGCCATCACCGCAGCGCTCTGACC | NR_002745 |
