# Supplementary material for: Potentiality of a triple microRNA classifier: miR-193a-3p, miR-23a and miR-338-5p for early detection of colorectal cancer
Source: BMC Cancer. 2013 Jun 8;13:280. doi: 10.1186/1471-2407-13-280 (PMC3691634; doi:10.1186/1471-2407-13-280)
Supplement: Additional file 2 — (A) List of significantly deregulated miRNAs (p<0.05) from tissue miRNA array. MiRNA expression was shown as fold change of cancer tissue versus adjacent normal mucosa. Positive value denotes up-regulation and negative value denotes down-regulation. (B) List of significantly deregulated miRNAs (p?<?0.05) from blood miRNA array. MiRNA expression was shown as fold change of TNM stages (I, II, III and IV) versus control samples. Positive value denotes up-regulation and negative value denotes down-regulation. [file 1471-2407-13-280-S2.doc]

**Additional file 2**

**(A)** **List of significantly deregulated miRNAs (*p* < 0.05) from tissue miRNA array.** MiRNA expression was shown as fold change of cancer tissue versus adjacent normal mucosa. Positive value denotes up-regulation and negative value denotes down-regulation.

| **MiRNA** | **Fold change** |
| --- | --- |
| hsa-let-7i-star | 1.76 |
| hsa-miR-105 | 2.13 |
| hsa-miR-10b | -1.79 |
| hsa-miR-10b-star | -1.86 |
| hsa-miR-1224-5p | 1.63 |
| hsa-miR-124 | -6.64 |
| hsa-miR-1244 | 1.77 |
| hsa-miR-1246 | 4.74 |
| hsa-miR-1247 | 8.74 |
| hsa-miR-1292 | 1.62 |
| hsa-miR-1296 | -1.63 |
| hsa-miR-1308 | 3.43 |
| hsa-miR-138 | -3.56 |
| hsa-miR-139-3p | -5.91 |
| hsa-miR-139-5p | -5.61 |
| hsa-miR-140-3p | -1.79 |
| hsa-miR-146b-3p | 2.34 |
| hsa-miR-148a | 1.82 |
| hsa-miR-149 | -2.41 |
| hsa-miR-150 | -1.61 |
| hsa-miR-181d | 1.63 |
| hsa-miR-182 | 3.64 |
| hsa-miR-183-star | 5.01 |
| hsa-miR-18a-star | 3.47 |
| hsa-miR-193a-3p | 8.72 |
| hsa-miR-195 | -2.03 |
| hsa-miR-198 | 1.54 |
| hsa-miR-203 | 3.54 |
| hsa-miR-20a | 1.94 |
| hsa-miR-215 | -3.21 |
| hsa-miR-21-star | 2.33 |
| hsa-miR-224 | 8.44 |
| hsa-miR-23a | 2.79 |
| hsa-miR-23b | 3.56 |
| hsa-miR-23b-star | -2.20 |
| hsa-miR-27b | -1.72 |
| hsa-miR-27b-star | -1.80 |
| hsa-miR-28-3p | -2.29 |
| hsa-miR-29b-2-star | -2.10 |
| hsa-miR-30a | -2.76 |
| hsa-miR-30a-star | -4.25 |
| hsa-miR-30c-1-star | -2.48 |
| hsa-miR-30c-2-star | -4.12 |
| hsa-miR-3162 | 1.90 |
| hsa-miR-338-5p | 8.20 |
| hsa-miR-342-3p | -1.59 |
| hsa-miR-378 | -2.81 |
| hsa-miR-378c | -3.15 |
| hsa-miR-378-star | -2.75 |
| hsa-miR-383 | -3.45 |
| hsa-miR-422a | -2.72 |
| hsa-miR-424-star | 2.20 |
| hsa-miR-429 | 2.03 |
| hsa-miR-431 | 1.75 |
| hsa-miR-483-3p | 1.63 |
| hsa-miR-493-star | 2.25 |
| hsa-miR-497 | -2.90 |
| hsa-miR-501-5p | 1.71 |
| hsa-miR-503 | 2.42 |
| hsa-miR-509-3p | 3.65 |
| hsa-miR-548x | -1.76 |
| hsa-miR-550-star | 2.57 |
| hsa-miR-552 | 8.89 |
| hsa-miR-622 | 8.66 |
| hsa-miR-663b | 5.40 |
| hsa-miR-767-5p | 2.62 |
| hsa-miR-887 | -2.02 |
| hsa-miR-940 | 1.58 |
| hsa-miR-941 | 2.65 |
| hsa-miR-96 | 1.86 |
| hsa-miR-99a | -1.57 |
| hsa-miR-99b | -1.85 |

**(B)** **List of significantly deregulated miRNAs (*p* < 0.05) from blood miRNA array.** MiRNA expression was shown as fold change of TNM stages (I, II, III and IV) versus control samples. Positive value denotes up-regulation and negative value denotes down-regulation.

| **MiRNA** | **Fold change** | | | |
| --- | --- | --- | --- | --- |
|  | **Stage I CRC** | **Stage II CRC** | **Stage III CRC** | **Stage IV CRC** |
| hsa-miR-122 | -1.90 | 1.89 | -1.18 | 7.34 |
| hsa-miR-122-star | 1.10 | -1.08 | 1.52 | -1.02 |
| hsa-miR-1245 | 1.55 | -1.01 | 1.12 | 1.08 |
| hsa-miR-1274b | 1.79 | -1.03 | -1.14 | 1.00 |
| hsa-miR-130a-star | 1.64 | -1.04 | -1.15 | -1.02 |
| hsa-miR-150 | -2.56 | -1.52 | -3.91 | -1.47 |
| hsa-miR-18b-star | 1.54 | -1.03 | 1.41 | 1.09 |
| hsa-miR-193a-3p | 1.01 | 1.21 | -1.00 | 1.85 |
| hsa-miR-2116-star | 2.21 | 1.13 | 1.17 | -1.05 |
| hsa-miR-23a | -1.05 | 1.55 | -1.07 | 1.45 |
| hsa-miR-23b | 1.58 | 1.51 | 1.41 | 1.74 |
| hsa-miR-296-5p | 1.88 | -1.05 | 1.31 | -1.07 |
| hsa-miR-3122 | -1.58 | 1.07 | -1.04 | -1.20 |
| hsa-miR-3183 | 2.22 | 1.00 | -1.13 | -1.16 |
| hsa-miR-338-5p | -1.32 | 1.74 | -1.04 | 2.40 |
| hsa-miR-342-3p | -1.37 | -1.30 | -1.77 | -1.25 |
| hsa-miR-409-5p | -1.28 | 1.08 | 1.18 | 1.66 |
| hsa-miR-4267 | -1.28 | -1.04 | 1.56 | 1.06 |
| hsa-miR-483-3p | -1.04 | 1.24 | 1.71 | 1.04 |
| hsa-miR-513a-3p | 1.64 | 1.06 | 1.24 | -1.01 |
| hsa-miR-520a-3p | 1.52 | -1.04 | -1.04 | -1.01 |
| hsa-miR-548u | 1.95 | -1.34 | -1.30 | -1.24 |
| hsa-miR-587 | 1.53 | 1.03 | 1.04 | -1.14 |
| hsa-miR-92a-2-star | 1.98 | -1.06 | -1.06 | -1.12 |
